# Semiconductor Quantum Dots for Biomedicial Applications

**DOI:** 10.3390/s111211736

**Published:** 2011-12-16

**Authors:** Lijia Shao, Yanfang Gao, Feng Yan

**Affiliations:** Jiangsu Affiliated Cancer Hospital with Nanjing Medical University, Jiangsu Institute of Cancer Prevention and Cure, Nanjing 210009, China; E-Mails: jia1987_2005@163.com (L.S.); gyfg05@yahoo.com.cn (Y.G.)

**Keywords:** quantum dots, bioanalysis, toxicology delivery, photodynamic therapy, cell imaging

## Abstract

Semiconductor quantum dots (QDs) are nanometre-scale crystals, which have unique photophysical properties, such as size-dependent optical properties, high fluorescence quantum yields, and excellent stability against photobleaching. These properties enable QDs as the promising optical labels for the biological applications, such as multiplexed analysis of immunocomplexes or DNA hybridization processes, cell sorting and tracing, *in vivo* imaging and diagnostics in biomedicine. Meanwhile, QDs can be used as labels for the electrochemical detection of DNA or proteins. This article reviews the synthesis and toxicity of QDs and their optical and electrochemical bioanalytical applications. Especially the application of QDs in biomedicine such as delivering, cell targeting and imaging for cancer research, and *in vivo* photodynamic therapy (PDT) of cancer are briefly discussed.

## 1. Introduction

**Figure 1.**
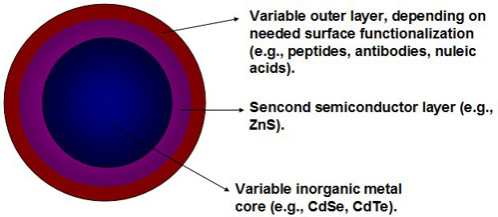
Schematic representation of a quantum dot. QDs are nanocrystals composed of a core of a semiconductor, usually composed of elements from groups II–IV, e.g., CdSe, or groups III–V, e.g., InP. The shell is typically a higher bandgap material such as ZnS. Finally, a capping outer layer such as silica can offer large-surface area for covalently linking to biorecognition molecules such as peptides, antibodies, nucleic acids and small-molecule ligands for further application. The diameter of QDs ranges between 2–10 nm.

Quantum dots (QDs) as colloidal nanocrystalline semiconductors have unique photophysical properties due to quantum confinement effects. They emit different wavelengths over a broad range of the light spectrum from visible to infrared, depending on their sizes and chemical compositions. Compared with the traditional organic fluorophores (e.g., organic dyes and fluorescent proteins), QDs have unique optical and electronic properties, such as larger absorption coefficients, size-tunable light emission, superior signal brightness, resistance to photobleaching and simultaneous excitation of multiple fluorescence colors [1–6]. In addition, the large-surface area of QDs is beneficial to covalently link to biorecognition molecules, such as peptides, antibodies, nucleic acids or small-molecule ligands for further application as fluorescent probes (Figure 1).

These properties of QDs herald a revolution from electronic materials science to biological applications [7]. Current and projected applications of QDs include using fluorescent labels for cellular labeling [1,8,9], intracellular sensors [10,11], deep-tissue and tumor targeting and imaging agents [7,12–17], sensitizers for photodynamic therapy (PDT) [18–21], vectors for gene therapy [22–26], magnetic resonance imaging (MRI) contrast agents [27,28] and so on. This review mainly summarizes the development of synthesis, the surface modification and toxicity of QDs, and briefly focuses on the application developments of QDs in the biomedical field.

## 2. The Surface Chemistry and Toxicity of QDs

Early in the 1990s, Bawendi and coworkers first reported a synthesis protocol for QDs with highly monodisperse, regular core structure and tunable particle size [29,30]. Up to now, the most successful and well-developed method to prepare highly luminescent II–VI QDs is the TOP/TOPO synthetic approach [31]. However, these QDs are insoluble in water, which limits their biological applications. Therefore, a number of surface functionalization studies have been developed to make QDs water-soluble and biologically compatible [29–37].

In one common approach, the original hydrophobic coatings are replaced by water-soluble functional molecules (e.g., dithiothreitol [38–40], mercaptocarbonic acids [41–44], 2-aminoethanethiol [33,45], dihydrolipoic acid [34–36,46,47], oligomeric phosphines [37,48], peptides [49–57], and cross-linked dendrons [58–61]) through the ligand exchange reactions. Because the optical properties of the inorganic core are often very sensitive to the surface, the ligand exchange process may result in poorer performance, particularly in the case of quantum dots [62].

The second approach is to encapsulate QDs in an amphiphile whose hydrophobic ends interleave with, but do not replace, the organic coating on QDs. This improvement for QDs synthesis is significant: (1) protecting the core/shell structure and maintaining the original photophysics of QDs; (2) making QDs water-soluble; (3) providing a biological interface and multiple functions [7]. However these kinds of QDs are not stable in biological settings because of relativelyweak anchoring of the single and double hydrophobic tails to the particle. Additionally, the hydrophilic end groups of even biocompatible surfactants may not protect nanocrystals from nonspecific biomolecular interactions [31]. Scientists have used amphiphilic polymers instead of simple amphiphile because single polymer chains can contain multiple hydrophobic units, their interactions with the native organic coatings on QDs can be numerous, and thus the encapsulant can be bound more strongly than conventional surfactants. However, the range of amphiphilic polymers for creating stable and nonaggregating QDs in biological settings has been relatively limited. Up to now, most of the amphiphilic polymers used are commercial and their hydrophobic/hydrophilic ratios are fixed, hence the cost is high and it may be different to control the process of forming water-soluble QDs and to optimize the forming conditions [31].

Although QDs have great prospects, the toxicity of QDs cannot be overlooked. During the processing of biological applications (e.g., cancer imaging, targeting and PDT treatment), the degradation products of QDs will do harm to the cells which they contact with, or produce immune responses with the components in blood [17]. The toxic degradation production routes are: first, the oxidation of the nanoparticle core/shell material can cause the release of free cadmium or other heavy metals, which will interrupt the normal cell activities [18]; secondly, the photosensitized production of reactive oxygen intermediates (ROI) also plays an important role in mediating the cell damage [63]; thirdly, the toxicity of capping materials should also be considered, several groups in capping materials such as mercaptoacetic acid and tri-*n*-octylphosphine oxide (TOPO) could produce toxicity to cells [12].

To reduce the cytotoxicity of QDs, replacement of the cadmium by nontoxic or less-toxic metals such as indium (In), or encapsulation of the core with a biocompatibile shell should be considered. Though In-based semiconducting dots contain arsenic, another toxin, the cytotoxicity of these dots may be small enough to keep the toxicity low. Fisher and coworkers [64] found that QDs could remain within the body for very long periods. Kim [8] reported that larger QDs generally accumulated in the reticuloendothelial system, such as the liver, spleen and lymphatic system for several months, but the size less than 5 nm could be removed by the kidney quickly. So in order to minimize the toxicity of QDs, QDs can be designed as smaller as they can, which can help them more easily to clean them out from the body.

In spite of the fact many investigators have paid close attention to and observed the side-effect of QDs, the definite metabolism of QDs *in vivo* remains uncertain [65–68]. Thus, it is still a necessary issue to investigate the detailed biochemical and pharmacological mechanism for further application of QDs in the human body.

## 3. Delivering QDs into Cells

Effective delivery of QDs into the targeted-cell is the primary requirement for the bioapplications of QDs [9,15,17,20,32,54]. It is a major step because if QDs cannot reach their site of action *in vivo*, they is useless. Furthermore, efficient delivery can also allow a reduction in dosage level, avoid non-specific side effects and reduce toxicity risks [66,69,70]. The current methods for delivering QDs into cells mainly include passive delivery, facilitated delivery and active delivery.

The general passive delivery for QDs is endocytosis, which is simple, without further functionalization of the QDs surface with a targeting ligand for uptake [66]. By incubating with the cells at appropriate concentration and exposure time, QDs will enter into cells though the nonspecific cell endocytosis. However, the nonspecific ingestion of this mode caused ineffective endosomal escape, and would impede the delivery of QDs to the cytoplasm or other organelles. Furthermore, high intracellular concentration of QDs can enhance the cytotoxicity in some cases [69].

**Figure 2.**
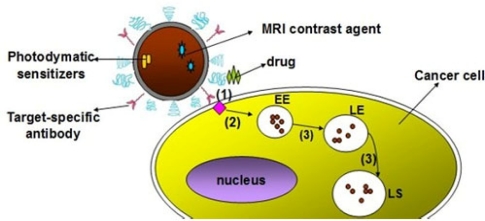
Schematic representation of delivering QDs into cells, the process comprises of three major stages: (**1**) endocytosis; (**2**) sequestering in early endosome (EE); (**3**) translocation to later endosomes (LE) or lysosomes (LS).

Facilitated delivery includes four ways: peptide-mediated uptake, protein-mediated delivery, polymer-mediated delivery and small molecule-mediated delivery [66]. Generally, these molecules are noncovalently assembled onto the surface of QDs for bioconjugation. Facilitated delivery could reduce the nonspecific absorption and side effects. However, QDs could also be uptaken by cell through endocytosis, leading to endosomal sequestration during the facilitated delivery strategies (Figure 2). As is well known, the high acidic of endosomes could degrade the QDs conjugates over time, thus free Polyethyleneimine (PEI) was used to encapsule the QDs conjugates to increase the stability [70]. Considering further application of cell imaging, more general endosomal escape strategies need to be developed in order to expand the application of facilitated delivery.

Active delivery is a direct physical manipulation of the cell by electroporation and microinjection. In comparison to facilitated delivery, QDs conjugates are delivered directly to the cytoplasm via electroporation by an endocytic pathway, without subsequent endosomal escape. However, the high cellular mortality rate and intracellar aggregation occurring during the delivery should be conquered [71]. Compared with electroporation, microinjection could deliver the QDs directly to the cytoplasm with lower cell death rate, and the rate of microinjection of QDs conjugates to cells depends on the physical constraints of cells, including morphology, membrane thickness, height, *etc*. [66]. Furthermore, this technology is very expensive. Therefore, considering the coexistence of advantages and drawbacks of the mentioned approaches, the appropriate way for delivering QDs into cell should be determined according to the specific experimental requirements. The relationship between the specific examples and the delivery strategies are listed in Table 1 [66].

## 4. QDs-Based Cancer Targeting and Imaging

**Figure 3.**
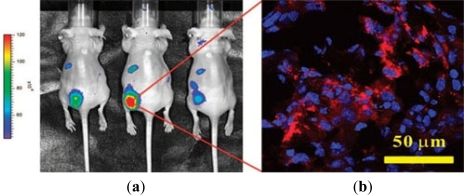
(**a**) Left panel: *In vivo* fluorescence imaging of three nude mice bearing MCF-7/HER2 xenografts implanted in the lower back 30 h after i.v. injection with anti-HER2 QD-ILs; (**b**) Right panel: A 5 μm section cut from frozen tumor tissues harvested at 48 h postinjection and examined by confocal microscopy by a 63× oil immersion objective (image size, 146 μm × 146 μm). The tumor section was examined in two-color scanning mode for nuclei stained by DAPI (blue) and QD-ILs (red). (Cited from Weng *et al.* [107]).

The photoluminescence (PL) of QDs is exceptionally bright and stable, making them potential candidates for biomedical imaging and therapeutic interventions. QDs conjugated with cancer specific ligands/antibodies/peptides were found to be effective for detecting and imaging human cancer cells. Gao and coworkers [67] firstly reported the QDs-antibody conjugates for *in vivo* targeting and imaging cancer, in which QDs-antibody conjugates were used as imaging probe for investigating and tracing QDs-PSMA antibody conjugates in mouse bearing subcutaneous human prostate cancer. It was found that the QDs-antibody conjugates were efficiently and uniformly distributed in prostate tumors due to the specific binding between PSMA antigen in prostate cancer cells and PSMA antibody on QDs. Cai and coworkers [105] conjugated NIR QDs with RED peptide, which could bind to the over-expressed αvβ3 integrin on the surface of U87MG glioblastoma cells and MDA-MB-435 human breast cancer cells to target cancer cell *in vivo*. By linking QDs to AFP (alpha-fetoprotein) antibody, an important marker for hepatocellular carcinoma cell lines, a specific immunofluorescent probes was obtained for further detection of AFP antibody in human serum. Yu *et al.* [106] demonstrated that the probe could target the specific hepatocellular carcinoma cells, and the expected results was obtained by investigating distribution of the probes in cancer cells by using a site-by-site measurement. Weng *et al.* [107] functionalized QDs with anti-HER2 scFv to synthesize the immunoliposome-based nanoparticles (QD-ILs). After incubating with HER2-overexpressing SK-BR-3 and MCF-7/HER2 cells, the QD-ILs exhibited efficient receptor-mediated endocytosis. *In vivo* fluorescence imaging showed that QD-ILs had localized prominently in tumors as well as in MPS organs (Figure 3). Liu *et al.* [68] reported a QDs-based wavelength-resolved spectral imaging for molecular mapping of tumor heterogeneity on human prostate cancer tissue specimens. By conjugating different QDs with specific protein biomarkers, such as E-cadherin, high-molecular-weight cytokeratin, p63, and α-methylacyl CoA racemase, structural distinct prostate glands and single cancer cells could be detected and characterized within the complex microenvironments of radical prostatectomy and needle biopsy tissue specimens using the wavelength-resolved spectral imaging.

The main advantage of QDs imaging is that it is non-ionizing and less hazardous [108]. In recent years, several groups have used QD probes for fluorescence immunostaining of fixed cells and tissue specimens [109–113]. QD-based immunohistochemistry (IHC) can improve both diagnostic sensitivity and specificity. In addition, because multiplexed QD staining can be carried out on intact cells and tissue specimens, it is expected to provide correlated molecular and morphological information, at the same time, this type of integrated biomarker and morphological data are not available from traditional analytical methods such as mass spectrometry, gene chips, protein microarrays, and polymerase chain reactions [109]. However, medical applications of QD-based IHC have achieved only limited success. A major bottleneck is the lack of robust protocols to define the key parameters and steps [109]. For example, there are no consensuses on methods for QD-antibody (QD-Ab) bioconjugation, tissue specimen preparation, multicolor QD staining, image processing and data quantification. So it is necessary to solve these problems, and let the QDs move further.

## 5. QDs Related Photodynamic Therapy for Cancer

Presently, the conventional types of cancer treatment (chemotherapy and radiation therapy), work by destroying fast-growing cells, but other types of fast-growing healthy cells (such as blood and hair cells) also can be damaged along with cancer cells, causing adverse reactions, or side effects. These side effects can range from fatigue and flu-like symptoms to hair loss and blood clotting problems. PDT developed in last century has become an FDA-approved therapy for different malignancies and with potential in other ailments such as coronary heart disease, AIDS and psoriasis [63].

**Figure 4.**
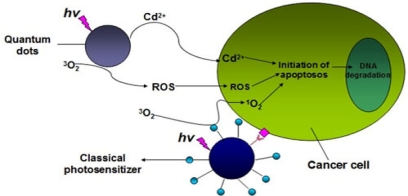
Schematic representation of possible mechanisms for induction of PDT processes by QDs and the classical photosensitizer.

Exploration of the use of light-activated drugs known as photosensitizers (PS) has been one of the most active areas of photomedical research in recent years [18–21,63,114,115]. PDT uses the combination of a photosensitizing drug and light in the presence of oxygen to cause selective damage to the targeting tissue. During PDT, reactive oxygen intermediates (ROI) is generated in the diseased cells by a simple and controllable light-activated process, which involves a photosensitizer that is capable of absorbing light appropriate wavelength and transfers energy or electron to oxygen or other molecules, and creates ROI such as singlet oxygen (^1^O_2_), hydroxyl radical (OH), super oxide anion (O_2_^−^) and hydrogen peroxide (H_2_O_2_). Then ROI will immediately react with vital biomolecules in cell organelles, leading to cell damage, mutation, death and photooxidation of cell constituents [19,20,63,114,115]. Singlet oxygen (^1^O_2_) is regarded as the main mediator of photo-induced cytotoxicity in PDT, which causes oxidation and degradation of cellular components, and ultimately cell apoptosis. [20,63,114,115] (Figure 4).

The standard PS drugs for PDT are porphyrin, phthalocyanines and chlorine derivatives. Porphyrin derivatives are the first generation photosensitizer. Despite the clinical success of porphyrin derivatives, some of their disadvantages like prolonged cutaneous photosensitivity, chemical impurity and weak absorption at therapeutic wavelengths have inspired the development of new PDT photosensitizers with improved optical and chemical properties. Phthalocyanines derivatives have favorable photophysical and chemical properties, which include strong absorbance at long wavelengths and chemical tunability through substituent addition on the periphery of the macrocycle or on the axial ligands. However, like most photosensitizing agents, these PS have poor solubility in water and tend to aggregate in aqueous solutions, which can result in loss of photochemical activity and affect their cell penetrating properties [63]. To resolve such issues nanoparticles are currently being explored as potential delivery systems for PDT photosensitizers or directly as PDT agents. The novel QDs-PS conjugates are used as a high ratio of PDT agents and anticancer targeting antibodies, where QDs can act as nanoscaffolds and solubilizers. They can also function as “energy-harvesting antenna” for PDT therapy due to their large one- or two-photon absorption cross-sections. Thus, QDs can be efficiently exited even deep within tissues and sensitized proximal PDT agents via energy transfer from QDs to PDT [21].

The novel QDs-PS conjugates showed many advantages over conventional PS drugs [17–21,63,114,115]: (1) they are species with well-defined size, shape, and composition, and can be synthesized by relatively simple and inexpensive methods; (2) they have been shown to be nontoxic in the absence of light but have the potential to be cytotoxic under irradiation; (3) they have photostability, and tunable and strong absorption, which can be tuned from the UV their composition and size; (4) the surface coating of QDs can be modified to enable them to become water soluble, biocompatible and target-specific.

However, researchers should be further investigated on the basis of predominances of the QDs-PS compared to the convention PS drugs. Despite many desirable properties of QDs for PDT, there still remain several important issues that need to be addressed to fully assess their applicability as PS in PDT. One major issue is the toxicity profile of the QDs inside the cells and their overall photostability once exposed to biological environments [63]. Another important matter that should be carefully investigated is how their surface composition affects the photosensitization process. Still, QDs-PS conjugates for cancer therapy are only suitable to superficial tumours is also need to be resolved [18].

## 6. Conclusions and Outlook

In the last decade, the unique photophysical properties and functions of QDs have been widely investigated, making them one of the most promising nanomaterials. Their outstanding performances such as high fluorescence yields, stability against photobleaching and the size-dependent luminescence features of QDs provide broad variety of applications for QDs in many fields. By acting as fluorescent, and photoelectrochemical as well as electrochemical probes, various QDs-based optical and electrochemical bioanalysis have already been successfully explored for sensing a wide range of molecules with high sensitivity and specificity. Furthermore, as a biomedical label, QDs can make a worthy contribution to the development of new diagnostic and delivery systems due to their unique optical properties. By combination of functional biomolecule-nanoparticle hybrid systems and the optical imaging and biophysics, QDs have been used as optical reporter units of biocatalytic transformations and can probe intracellular processes *in vitro*. QDs as a novel probe for *in vivo* analysis and clinic therapy such as PDT open an attractive new field with promising prospectives in biomedicine.

## Figures and Tables

**Table 1.** Selected strategies for the intracellular delivery of QDs.

**Strategy**	**Mechanism**	**Examples**	**Targeted Cells**	**References**
Passive uptake	Electrostatic interactions	-	HeLa	[15]
			Human macrophages	[27,72]
			Breast cancer (MDA-MB-231)	[73,74]
			Human melanoma cells (LU1205)	[75]

Facilitated delivery	Peptide-mediated	TAT	Human embryonic kidney	[76]
			HeLa	[77]
			Mesenchymal stem cells	[53]
			Jurkat cells	[28]
		Pep-1 (Chariot)	Osteoblast	[78]
			Vascular endothelial cells	[78]
		RGD motify	Fibroblast (NIH 3T3)	[79]
			Epidermoid carcinoma	[80]
	Protein-mediated	Neuropeptide	HeLa	[17]
		Transferrin	Human pancreatic cancer	[2,81,82]
		Antibody	Breast cancer (MCF-7)	[11]
		EGF	Mesenchymal stem cells	[83]
			Chinese hamster ovary	[84–86]
			Medulloblastoma tumors	[87]
			Glioma tumors	[87]
		Cholera toxin B	Fibroblast	[88,89]
		NGF	PC12 neural cells	[90,91]
	Polymer/lipid-mediated	Lipid polymers	Mouse lymphoma	[92]
			HeLa	[93]
			A549 epithelial lung HeLa	[94]
		Polyethyleneimine	HeLa	[95]
	Drug-mediated	Tiopronin	Fibroblast	[96]
	Small molecule	Glucose/sugar	*S. cerevisiae* (Baker’s yeast)	[97,98]
		Folate	Epidermal carcinoma	[99]
		Adenine/AMP	Bacteria (*Bacillus subtilis*, *E. coli*)	[100,101]
		Dopamin	A9 mouse fibroblast with transfected dopamine receptor	[102]

Active Delivery	Electroporation	-	HeLa	[71]
			Mouse neural stem progenitor cells	[9]
	Microinjection	-		[71]
			Xenopus embryo	[103]
			HeLa	[104]
			Human embryonic kidney	
